# Differential Cytotoxicity of Acetaminophen in Mouse Macrophage J774.2 and Human Hepatoma HepG2 Cells: Protection by Diallyl Sulfide

**DOI:** 10.1371/journal.pone.0145965

**Published:** 2015-12-29

**Authors:** Haider Raza, Annie John

**Affiliations:** Department of Biochemistry, College of Medicine and Health Sciences, UAE University, PO Box 17666, Al Ain, United Arab Emirates; Wayne State University School of Medicine, UNITED STATES

## Abstract

Non-steroidal anti-inflammatory drugs (NSAIDs), including acetaminophen (APAP), have been reported to induce cytotoxicity in cancer and non-cancerous cells. Overdose of acetaminophen (APAP) causes liver injury in humans and animals. Hepatic glutathione (GSH) depletion followed by oxidative stress and mitochondrial dysfunction are believed to be the main causes of APAP toxicity. The precise molecular mechanism of APAP toxicity in different cellular systems is, however, not clearly understood. Our previous studies on mouse macrophage J774.2 cells treated with APAP strongly suggest induction of apoptosis associated with mitochondrial dysfunction and oxidative stress. In the present study, using human hepatoma HepG2 cells, we have further demonstrated that macrophages are a more sensitive target for APAP—induced toxicity than HepG2 cells. Using similar dose- and time-point studies, a marked increase in apoptosis and DNA fragmentation were seen in macrophages compared to HepG2 cells. Differential effects of APAP on mitochondrial respiratory functions and oxidative stress were observed in the two cell lines which are presumably dependent on the varying degree of drug metabolism by the different cytochrome P450s and detoxification by glutathione S-transferase enzyme systems. Our results demonstrate a marked increase in the activity and expression of glutathione transferase (GST) and multidrug resistance (MDR1) proteins in APAP-treated HepG2 cells compared to macrophages. This may explain the apparent resistance of HepG2 cells to APAP toxicity. However, treatment of these cells with diallyl sulfide (DAS, 200 μM), a known chemopreventive agent from garlic extract, 24 h prior to APAP (10 μmol/ml for 18h) exhibited comparable cytoprotective effects in the two cell lines. These results may help in better understanding the mechanism of cytotoxicity caused by APAP and cytoprotection by chemopreventive agents in cancer and non-cancerous cellular systems.

## Introduction

Acetaminophen-induced toxicity, like many other drugs, may have a great deal of variations at the molecular, cellular, tissue, organ and organism levels [1–2]. Metabolic alterations and increased oxidative stress is considered to be the key aspects of hepatotoxicity and apoptotic as well as necrotic cell death by acetaminophen (APAP) [3–5]. The initial events in APAP-induced toxic injury lead to the activation of a secondary innate immune response by up regulation of proinflammatory cytokines and inflammasome [6–7]. Thus, alterations in the microenvironment by macrophages and their chemical communication and coordination with tissues play a major role in the progression and prevention of drug-induced toxicities and tissue repair. The precise molecular mechanism of APAP cytotoxicity however, is still controversial [8–9]. Reports suggest glutathione (GSH) depletion, oxidative stress and mitochondrial dysfunction in APAP-induced toxicity [3,10–11].The general consensus in APAP-induced toxicity is that the drug is mainly metabolized by various cytochrome P450s such as CYP2E1, CYP3A4, CYP1A2 and CYP1A1 to its active metabolite, mainly N-acetyl-p-benzoquinone imine (NAPQI) [12–13] which conjugates with GSH causing depletion of cellular GSH pools and increase in oxidative stress. Studies have suggested that APAP toxicity exhibited a biphasic response in which the metabolism of APAP is responsible for initial toxicity followed by mitochondrial dysfunctions [9,14–19]. The selective inhibition of proinflammatory signaling and induction of autophagy which removes damaged mitochondria, attenuates APAP-induced liver toxicity [7, 18, 20–21].

Both cytotoxic and cytoprotective effects of macrophages have been reported in APAP-induced toxicity [19, 22–23]. Our previous study on J774.2 macrophages demonstrated that APAP induces cytotoxicity and apoptosis by increasing ROS production, depletion of GSH pool, increase in oxidative stress and mitochondrial dysfunction [24–25]. Using macrophages and HepG2 cells as in vitro models, we have recently reported that aspirin treatment also induces oxidative stress and mitochondrial dysfunction, albeit at different levels [26–29].

Induction of cellular resistance has also been reported after APAP treatment. Several studies suggest that APAP treatment may also develop resistance towards drug toxicity by altering multidrug resistance protein, JNK-dependent signaling, autophagy in cells under in vitro conditions and in vivo in mice [20, 30–31]. There are multiple metabolic factors which determine the APAP-induced initial or long term cytotoxicity, which may or may not be dependent upon GSH depletion, metabolic activation and detoxification of the drug by the enzymes. However, APAP toxicity in vivo and in vitro has been correlated with CYP450s enzymes, particularly with CYP2E1 and CYP3A4 which metabolizes APAP to its toxic metabolites [13, 32] and could be blocked by CYP2E1 modulators such diallyl sulfide (DAS), a major garlic constituent, and antioxidants such as N-acetylcysteine [17,33–34].The objective of the present study was to elucidate the molecular mechanisms of differential toxicity of APAP in HepG2 cells and macrophages and the protection of cytotoxicity by DAS. The main focus of our study is to highlight the role of drug metabolizing enzymes, glutathione metabolism, oxidative stress and mitochondrial function in APAP induced cytotoxicity and cytoprotection by DAS.

## Materials and Methods

### Reagents

N-Acetyl-p-aminophenol (acetaminophen, APAP), diallyl sulfide (DAS), 5,5’-dithio bis-2-nitrobenzoic acid (DTNB), NADPH, glutathione reductase, 1-chloro 2,4-dinitrobenzene (CDNB), oxidized glutathione (GSSG) and reduced glutathione (GSH), N-ethyl malleimide (NEM), ethacrynic acid, 4-hydroxynonenal (4-HNE), cumene hydroperoxide, dimethylnitrosamine (DMNA), erythromycin, Coenzyme Q2, succinate, cytochrome c, ethoxyresorufin and methoxyresorufin and ATP bioluminescent assay kit were purchased from Sigma-Aldrich Fine Chemicals (St Louis, MO, USA). Apoptosis detection kit for flow cytometry was from BD Pharmingen (BD Biosciences, San Jose, USA) and comet assay kits were procured from Cell Biolabs, Inc. (San Diego, CA, USA). Kits for mitochondrial membrane potential were purchased from R&D Systems, (MN, USA). 2’, 7’-dichlorofluorescein diacetate (DCFDA) was purchased from Molecular Probe (Eugene,OR,USA). Aconitase assay kit was procured from Oxis International Inc. (Portland, OR, USA). HepG2 cells were purchased from American Type Culture Collection (Manassas, VA, USA) and murine macrophage J774.2 cells were purchased from European Collection of cell cultures (Health Protection Agency Culture Collections, Salisbury, UK). Antibodies against microsomal GST and GST A4-4 were generous gifts from Prof. Ralf Morgenstern, Karolina Institute, Stockholm, Sweden and Prof. Bengt Mannervic, Uppsala University, Uppsala, Sweden, respectively. Polyclonal antibodies against CYP1A1, CYP1A2, CYP2E1 and CYP3A4 were purchased from Amersham Int. Plc. (Amersham, UK) and GSTpi specific isoenzyme from Biotrin (Dublin, Ireland). MDR1and β-actin antibodies were procured from Santa Cruz Biotechnology, Inc, (CA, USA). Reagents for cell culture and for SDS-PAGE and Western blot analyses were purchased from Gibco BRL (Grand Island, NY, USA) and from Bio Rad Laboratories (Richmond, CA, USA) respectively.

### Cell Culture, Treatment and Fractionation

Macrophage J774.2 cells and HepG2 cells were grown in poly-L-lysine coated 75 cm^2^ flasks (~2.0–2.5 x10^6^ cells/ml) in DMEM medium supplemented with 10% heat inactivated fetal bovine serum in the presence of 5% CO_2_-95% air at 37°C. Cells were treated with APAP (10 μmol/ml) for 18 hours after treatment with or without 200 μM DAS for 24 h. After the desired time of treatment, cells were harvested, washed with PBS (pH 7.4) and homogenized in H-medium buffer (70 mM sucrose, 220 mM mannitol, 2.5 mM HEPES, 2 mM EDTA, and 0.1 mM phenylmethylsulfonylfluoride, pH7.4) at 4°C. Mitochondria and postmitochondrial (PMS) fractions were prepared by centrifugation and the purity of the isolated fractions for cross contamination was checked as described before [24–28]. Control cells were treated with vehicle alone. The choice of time and doses were based on our previous publications and literatures using acetaminophen in these cell lines as well as MTT viability test [24–28].

### DNA Fragmentation, Apoptosis, ROS Assays

#### DNA fragmentation

DNA fragmentation was assayed by UV transillumination after staining the electrophoretically (by 1.5% agarose gel) separated fragments with 0.5 μg/ml ethidium bromide as described before [24–28].

#### Comet assay

Cellular DNA damage was assessed using the single cell gel electrophoresis or comet assay according to the vendor’s protocol. Briefly, following treatment, cells were washed in cold PBS, centrifuged and resuspended at 1x10^5^ cells/ml in cold PBS. An aliquot of the resuspended cells was combined with low melting point agarose at 1:10 ratio, mixed and this cell suspension transferred to 3-well glass slides, ensuring complete well coverage and kept in the dark for 15 min at 4°C. Slides were then incubated for 1h at 4°C in lysis buffer. Following lysis, the slides were placed in 1.2% NaOH solution for 30 min at 4°C. The slides were then transferred to a horizontal electrophoresis chamber containing electrophoresis buffer (300mM NaOH, 1mM EDTA, pH>13) and run for 15–30 min at 1volt/cm. The slides were then washed with distilled water, followed by 70% ethanol and then stained using the Vista Green DNA dye at room temperature for 15 min. The slides were then analyzed using an Olympus fluorescence microscope. Typical representation from 3 such experiments has been shown.

#### Apoptosis

The apoptosis assay by flow cytometry using Annexin V conjugated FITC and propidium iodide was performed according to the vendor’s protocol as described in the previous study [24–26]. The apoptotic cells were estimated by the percentage of cells that stained positive for Annexin V-FITC.

#### ROS assay

The intracellular production of ROS was measured by using lucigenin-coupled chemiluminiscence assay which preferentially measures superoxides as described before [24–28].

### Measurement of GSH Metabolism

Total cellular GSH concentration was measured by enzymatic recycling of oxidized glutathione by Griffith’s method using NADPH and GSH-reductase as described before [24–28].Total GST activity was measured in APAP treated cells by using CDNB as substrate [24–28]. Substrate specific GST pi and GST A4-4 activities were measured using ethacrynic acid and 4-hydroxynonenal (4-HNE) as substrates. Microsomal GST activity was measured in the presence of NEM using CDNB as substrate. GSH-peroxidase activity was measured using cumene hdroperoxide as substrate as described before [27–28].

### Measurement of CYP450 Activities

Post-mitochondrial supernatant was used to measure the activities of the enzymes,CYP1A1,CYP1A2, CYP2E1 and CYP3A4, using ethoxyresorufin, methoxyresorufin, DMNA and erythromycin, respectively, as substrates as described before [25,28, 35].

### Measurement of ATP Level

The ATP content in the cell lysate was determined using an ATP Bioluminescent cell assay kit according to the manufacturer’s suggestion and samples were read using the TD-20/20 Luminometer (Turner Designs, Sunnyvale, CA). A standard curve for different concentrations of ATP (5–500 nM) was used to calculate the concentration of ATP in the control and treated cells.

### Measurement of Mitochondrial Membrane Potential (Δψm)

The mitochondrial membrane potential (Δψm) was measured by flow cytometry using a fluorescent cationic dye according to the vendor’s protocol (DePsipher^TM^, R &D System Inc.). DePsipher has the property of aggregating upon membrane polarization forming an orange-red fluorescent (absorption/emission 585/590nm) compound. If the membrane potential is reduced, the dye cannot access the transmembrane space and remains in its green fluorescent (510/527nm) monomeric form.

### Measurement of Enzymes of Krebs Cycle and Mitochondrial Respiratory Complexes

The freshly isolated mitochondria (5μg protein) from control and treated macrophage and HepG2 cells were suspended in 1.0 ml of 20 mM potassium phosphate buffer, pH 7.4, in the presence of the detergent, lauryl maltoside (0.2%). Mitochondrial matrix enzyme aconitase activity was measured by NADPH coupled conversion of citrate to isocitrate in the presence of isocitrate dehydrogenase using Bioxytech Aconitase-340 assay kit as described before [26]. NADH-ubiquinone oxidoreductase (Complex I), and cytochrome c oxidase (complex IV) were measured using the substrates ubiquinone and reduced cytochrome c, respectively, as described before [24–28].

### SDS-PAGE and Western Blot Analysis

Protein (50 μg/well), from the sub-cellular fractions prepared as described above, was separated on 12% SDS-PAGE according to the method of Laemmli (1970) [36]. Electrophoresed proteins were transferred to nitrocellulose membrane and subjected to Western blotting [37]. Transferred proteins were then probed with rabbit antibodies against CYP2E1, CYP3A4, CYP1A1 and CYP1A2 (1:1000 dilution), GST A4-4, pi, microsomal GST1-1 (1:1000 dilution), and subsequently detected with peroxidase conjugated species specific secondary antibodies (1:5000 dilution). The signals were visualized and further densitometric analysis performed using the Typhoon FLA 9500 system (GE Healthcare, Uppsala, Sweden) and expressed as relative intensity (R.I) compared to the untreated control. β-Actin was used as a loading control. [24–28].

### Statistical Analysis

Statistical comparison of control and drug treated groups was analyzed using SPSS software (version 21) by ANOVA followed by Dunnett’s post-hoc analysis. The values are expressed as mean ± SEM and p values≤0.05 were considered significant.

## Results

### Effect of APAP on DNA Fragmentation, Apoptosis, and ROS Production

**Fig 1:**
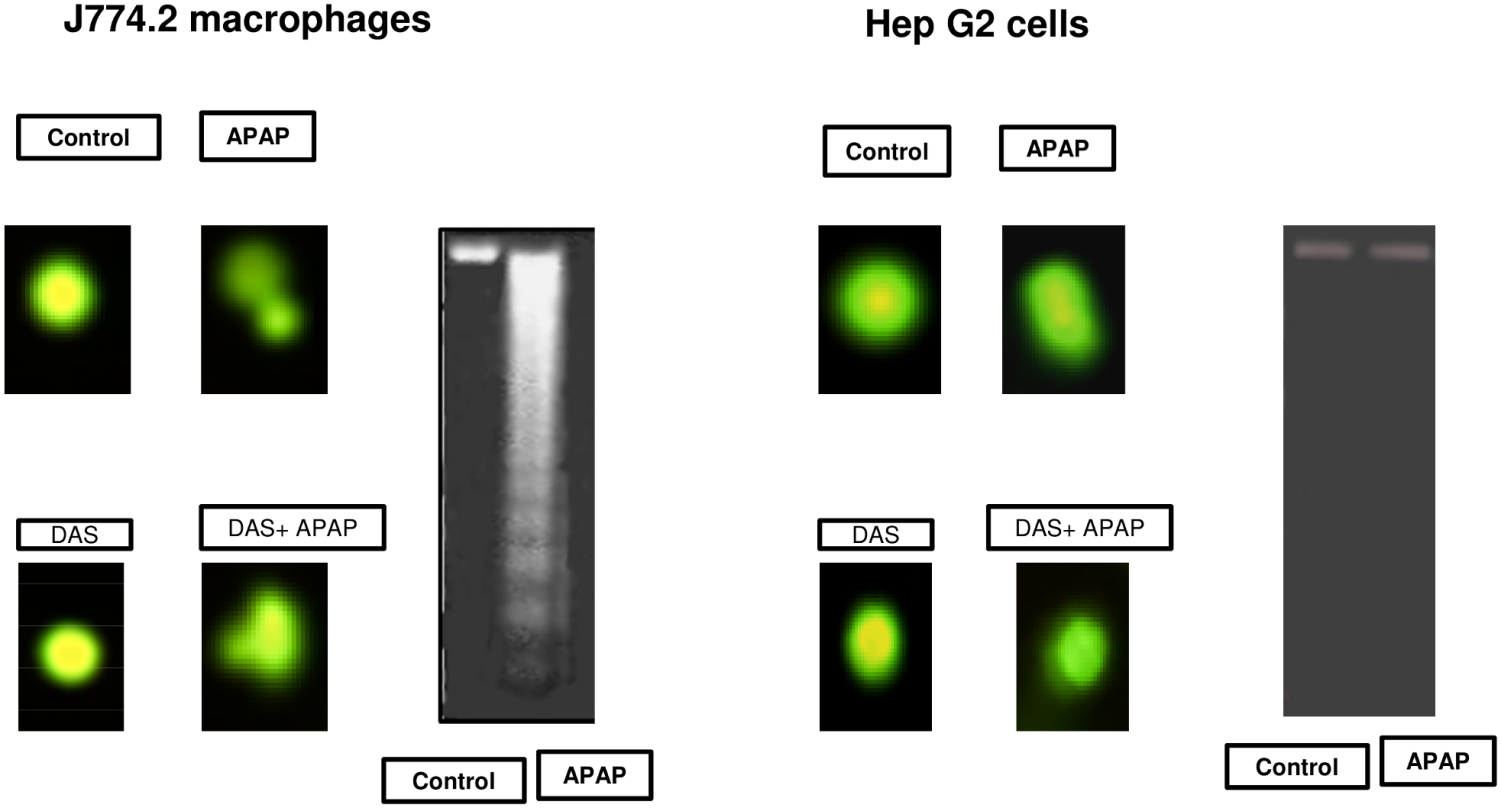
APAP-induced DNA fragmentation. J774.2 macrophages and HepG2 cells were cultured and treated with APAP (10 μmol/ml) for 18 hours after treatment with or without 200 μM DAS for 24 h as described in Materials and Methods. DNA fragmentation was visualized by 0.5μg/ml ethidium bromide staining of DNA fragments separated on 1.5% agarose gel. DNA breakdown was also visualized by using a single cell comet assay according to the vendor’s protocol. The slides were examined at x100 magnification using an Olympus fluorescence microscope. Images of 50 randomly selected nuclei were analyzed per slide. Typical results from 3 such experiments have been shown.

Fig 1 shows that the macrophages are much more sensitive to APAP treatment than HepG2 cells as indicated by the fragmentation and laddering of DNA in these cells. Comet single cell gel electrophoresis assay showed enhanced DNA fragmentation in macrophages after treatment with APAP (10 μmol/ml) for 18 h). No significant DNA fragmentation or laddering was however observed in HepG2 cells under these conditions.

**Fig 2:**
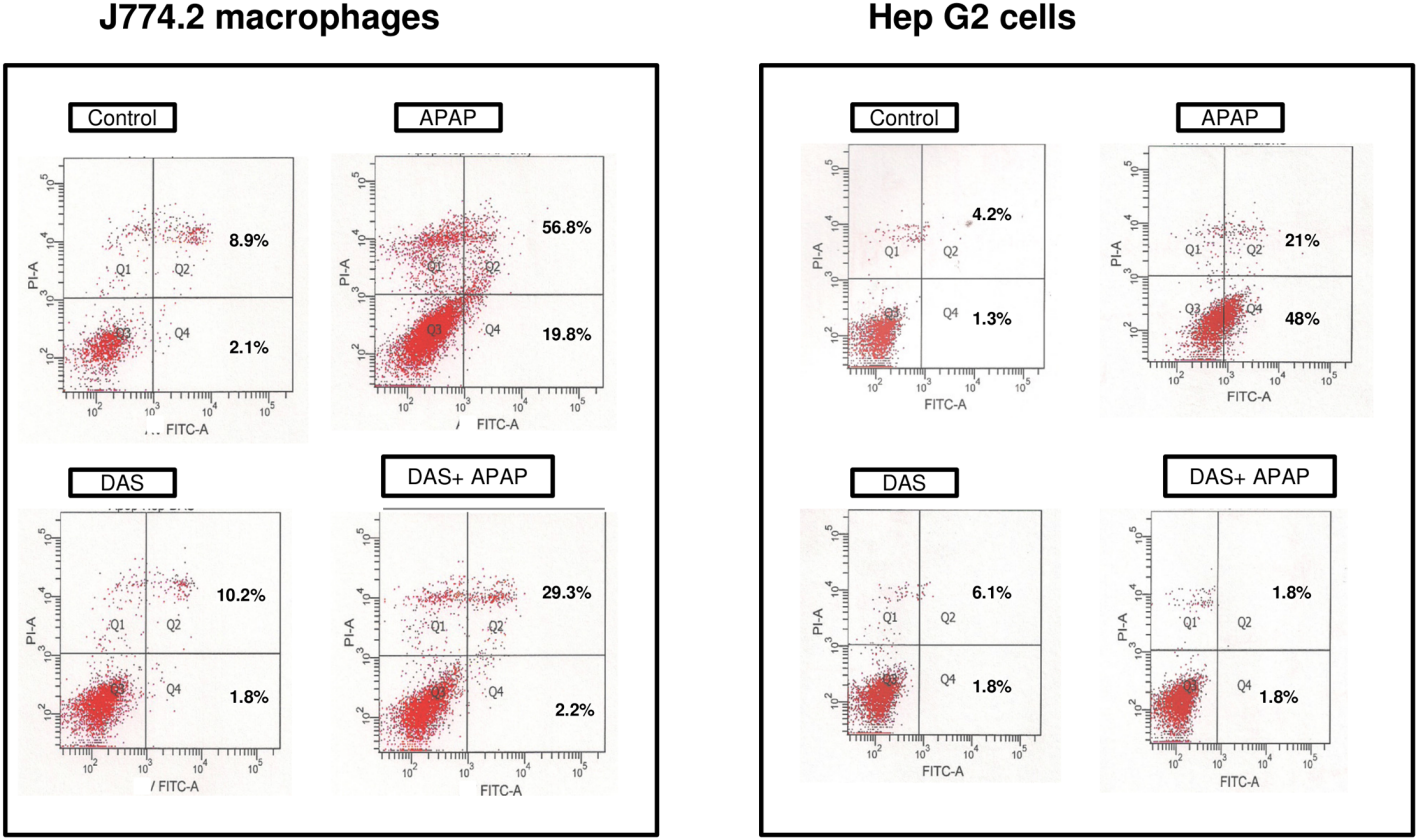
APAP-induced apoptosis. Apoptosis in J774.2 macrophages and HepG2 cells were measured after APAP and DAS treatment using Flow cytometry as described in the vendor’s protocol using the Becton Dickinson FACSCantoII analyzer. Apoptotic cells were estimated by the percentage of cells that stained positive for Annexin V-FITC. Representative histograms of flow cytometric results are shown.

Similarly, more cell death was observed in macrophages than in HepG2 cells after APAP treatment (Fig 2 right upper panel, 56.8% vs 21%) when compared with control untreated cells. Interestingly, HepG2 cells exhibited a higher level of early apoptosis than that seen in macrophages (Fig 2 right lower panel, 48% vs 19.8%). These results suggest more late apoptosis (necrotic) cell death in macrophages by APAP than in HepG2 cells. These results confirm our earlier electron microscopic studies on macrophages treated with different doses of APAP at different time points [25]. Our results also show that DAS treatment resulted in a significant reduction of apoptosis in both the cell systems. However, the protective effect of DAS treatment on necrotic cell death induced by APAP was more apparent in HepG2 cells than in macrophages. These results suggest that macrophages are more sensitive to APAP induced toxicity than HepG2 cells.

**Fig 3:**
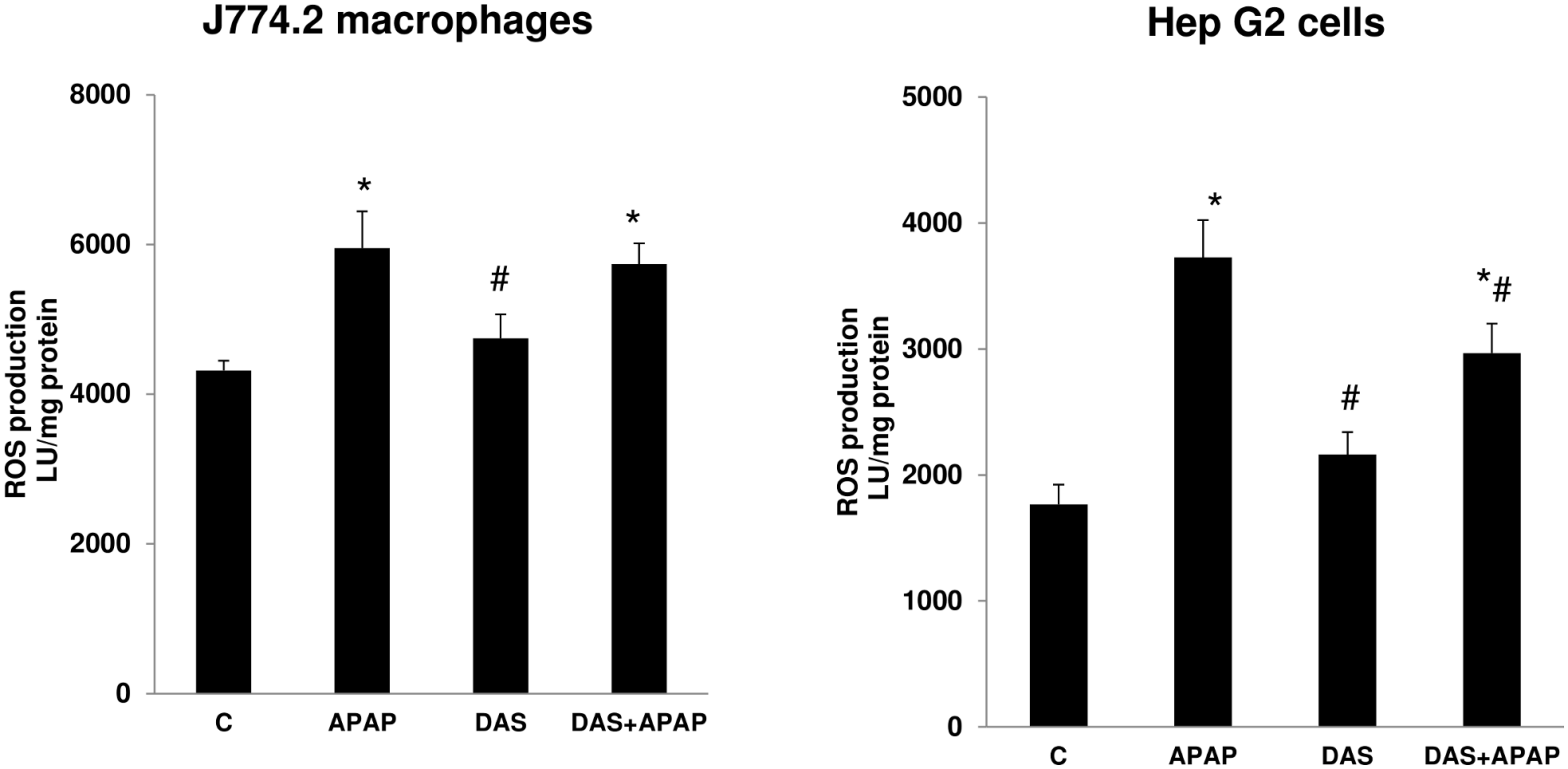
Effect of APAP on ROS production. Intracellular production of ROS was measured using lucigenin coupled method and chemiluminescence was measured using Turner’s luminometer as described in Materials and Methods [24–28]. The values expressed are mean ±SEM for at least three determinations. Asterisks (*) indicate significant difference (p ≤0.05) from control values, # indicate significant difference (P ≤0.05) from APAP-treated group.

APAP treatment in macrophages and HepG2 cells has significantly increased ROS production (Fig 3). Pretreatment with DAS significantly reduced the level of ROS production in APAP treated HepG2 cells but not in macrophages. DAS alone does not cause any appreciable alterations in ROS production in these cell lines.

### Effect of APAP on GSH Metabolism

**Fig 4:**
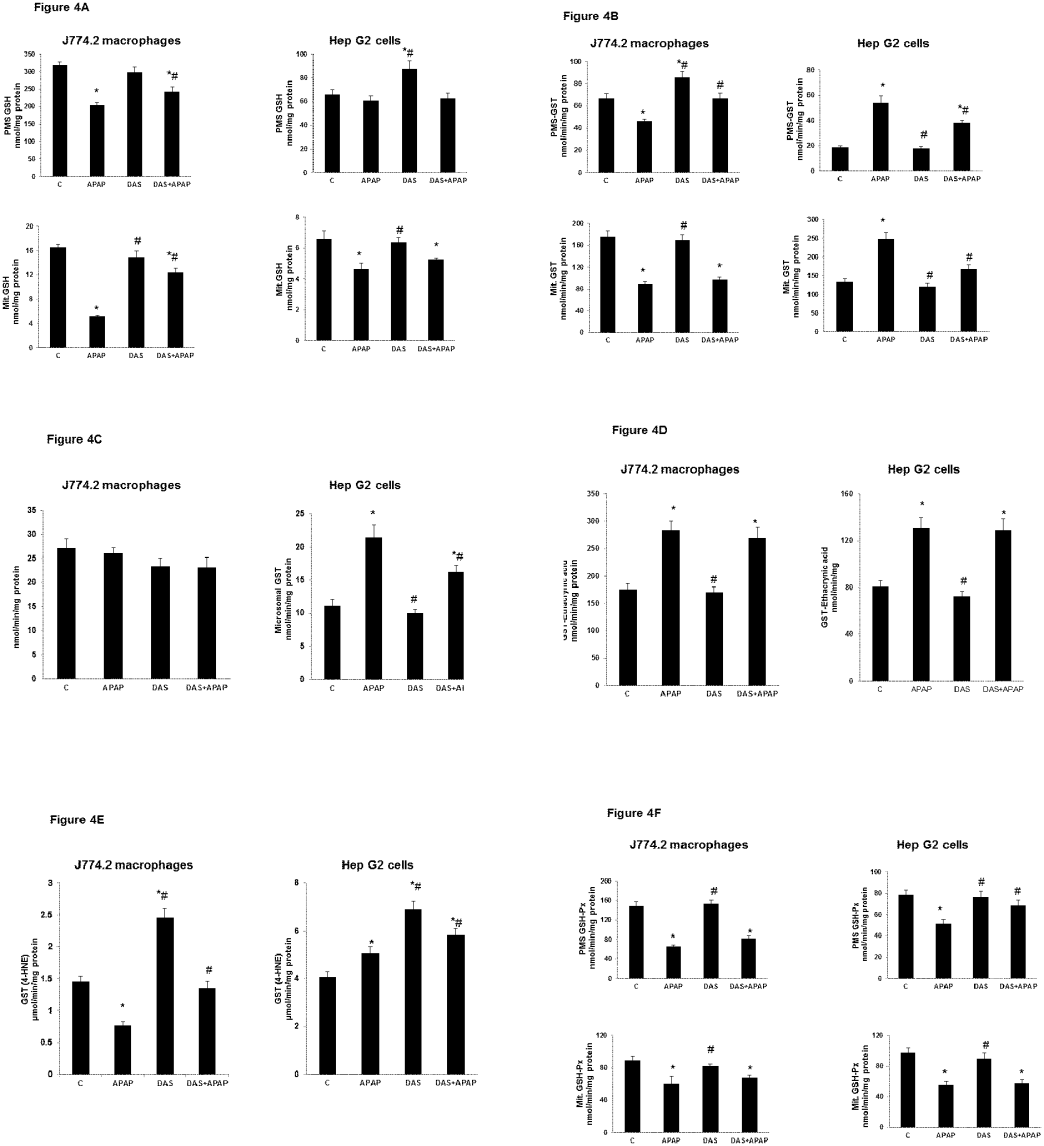
Effect of APAP on GSH metabolism. J774.2 macrophages andHepG2 cells were treated with APAP and DAS alone or in combination and GSH levels in the mitochondria and post-mitochondrial supernatant (PMS) were measured by enzymatic method as described in the Materials and Methods (4A). Total GST-conjugating activity was measured using CDNB as a substrate (4B). Microsomal GST activity was measured using CDNB as a substrate in the presence of NEM as an activator of membrane-bound microsomal GST (4C). Ethacrynic acid was used as substrate to measure GSTpi isoenzyme (4D) and 4-HNE was used to measure GSTA4-4 isoenzyme (4E) as described before [24–28]. GSH-Px activity was measured using cumene hydroperoxide as a substrate (4F). Results are expressed as mean ± SEM of three determinations. Asterisks (*) indicate significant difference (p ≤0.05) from control values, # indicate significant difference (p ≤0.05) from APAP-treated group.

A marked decrease in total GSH level in the mitochondrial compartment and cytosolic fraction was observed in macrophages after APAP treatment (Fig 4A). GSH concentration was reduced significantly only in the mitochondrial compartment of HepG2 cells (Fig 4A). DAS treatment exhibited a significant recovery in the concentration of GSH. However the concentration of mitochondrial GSH was still below the control untreated cells. These results again suggest that the macrophages are more sensitive to APAP- induced cytotoxicity than HepG2 cells.

Interestingly, differential GSH-CDNB conjugating activity of glutathione S-transferase enzyme was observed in the macrophages and HepG2 cells treated with APAP (Fig 4B). While a significant decrease in enzyme activity was observed in the cytosolic and mitochondrial compartments in macrophages, a significant increase in total GSH-CDNB conjugating activity was observed in HepG2 cells. Pretreatment with DAS brought the enzyme activity close to that of control untreated cells. On the other hand, membrane bound microsomal GST was only significantly increased in HepG2 cells after APAP treatment but not in macrophages (Fig 4C).

In order to further investigate the role of specific GST isoenzymes in GSH-conjugation in these cell lines, we further studied the enzyme activity using isoenzyme specific substrates, ethacrynic acid for GST pi and 4-hydroxynonenal (4-HNE) for GST A4-4. GST pi activity was increased in APAP- treated macrophages as well as HepG2 cells (Fig 4D). On the other hand, 4-HNE-conjugating GST A4-4 activity was slightly increased in HepG2 cells, while a marked reduction in enzyme activity was observed in the macrophage cells (Fig 4E). This was also confirmed by SDS-PAGE/Western blot analysis of enzyme protein expression using isoenzyme-specific antibodies (described later). DAS treatment alone had little effect on GST pi activity in these cells. However, GST A4-4 activity was enhanced in both cell lines. These results suggest the differential responses of macrophages and HepG2 cells towards APAP- induced cytotoxicity.

GSH-peroxidase (GSH-Px) activity, on the other hand, was significantly inhibited in the mitochondrial and post-mitochondrial compartments after APAP treatment both in the macrophages as well as in HepG2 cells (Fig 4F). DAS treatment resulted in partial recovery of enzyme activity only in the post-mitochondrial compartment of HepG2 cells.

### Effects of APAP on CYP450 Activities

**Fig 5:**
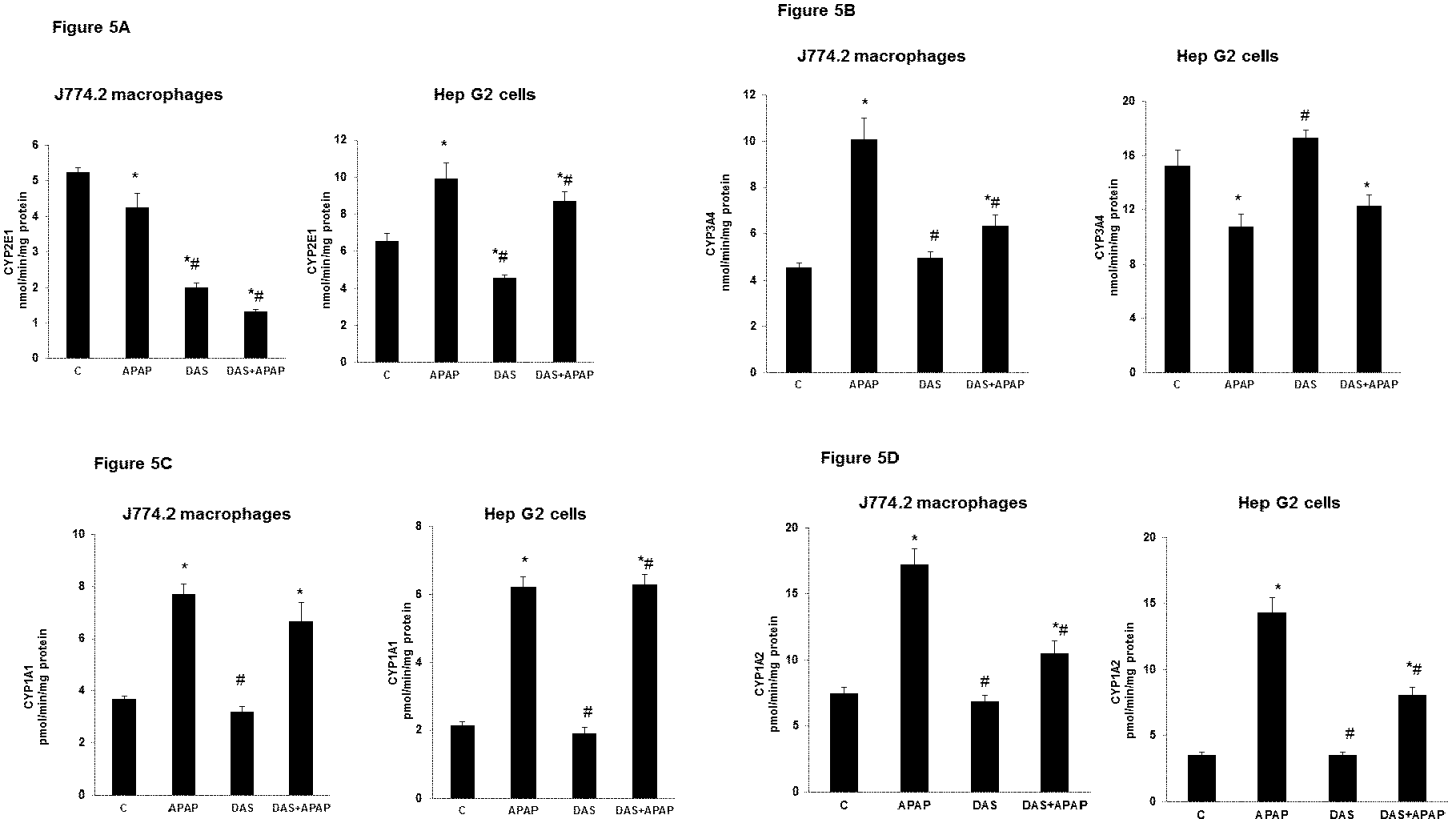
Effect of APAP on CYP450 activities. J774.2 macrophages and HepG2 cells were treated with APAP and DAS as described above and post mitochondrial supernatant was used to measure CYP2E1 (5A), CYP3A4 (5B), CYP1A1 (5C) and CYP1A2 activities as described in the Materials and Methods. The values expressed are mean ±SEM of three determinations. Asterisks (*) indicate significant difference (p≤0.05) from untreated control cells, # indicate significant difference (p≤0.05) from APAP-treated group.

We used isoenzymes-specific substrates to measure the microsomal activities of CYP2E1, CYP3A4, CYP1A1 and CYP1A2 in macrophages and HepG2 cells treated with APAP and DAS. While CYP2E1 activity was significantly lower in the macrophages, the enzyme activity was markedly (56%) increased in HepG2 cells (Fig 5A). As expected, DAS treatment in the macrophages resulted in a 2–3 fold decrease in activity. However, unlike in the macrophages, the enzyme activity remained higher than that seen in the control untreated HepG2 cells (Fig 5A).

Interestingly, alterations in CYP3A4 activity exhibited a different pattern. A marked increase (about 2-fold) in enzyme activity was only observed in the macrophages while a significant decrease (30%) was observed in HepG2 cells (Fig 5B). These results have further confirmed our earlier observation on the alterations of different CYPs in macrophages and HepG2 cells [25].

CYP1A1 and CYP1A2 activities in both the cell lines have demonstrated a more or less similar pattern of alterations (Fig 5C and 5D). A 2–3 fold increase in enzyme activities was observed after APAP treatment in both the cell lines. DAS treatment, either alone or in the presence of APAP, had no significant effects on CYP1A1enzyme activity. CYP1A2 activity was, however, significantly reduced after DAS treatment in APAP-induced macrophages and HepG2 cells (Fig 5D).

### Effect of APAP on Mitochondrial Functions: ATP Production, Membrane Potential and Respiratory Enzymes Complexes

**Fig 6:**
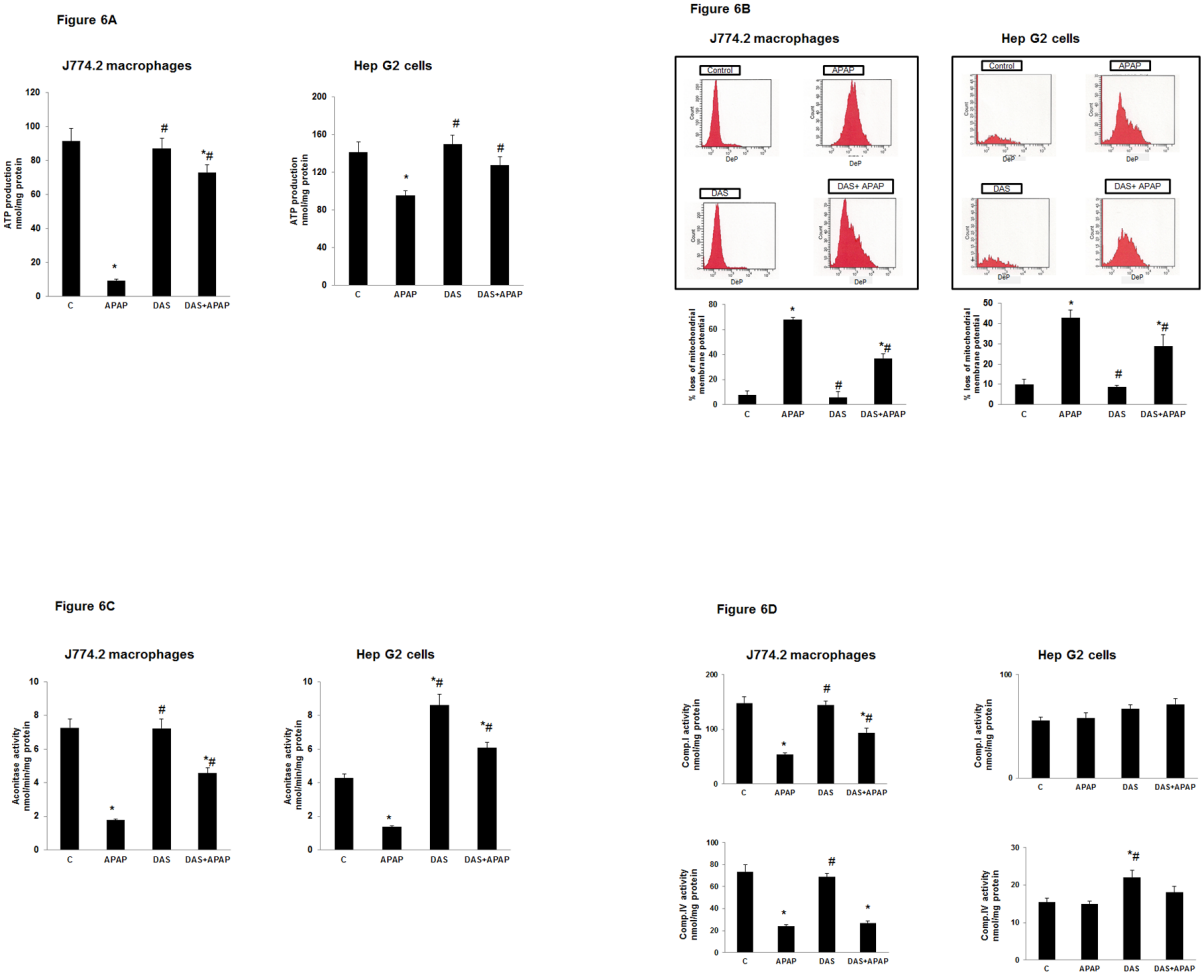
Effect of APAP on ATP production, membrane potential, mitochondrial matrix enzyme, aconitase and mitochondrial respiratory complexes. APAP treated cells were lysed and ATP was measured by the luciferase-dependent chemiluminescence assay as described in the vendor’s protocol (6A). The mitochondrial membrane potential was measured by using a mitotracker fluorescent cationic dye according to the manufacturer’s protocol (6B). % reduction in mitochondrial membrane potential is shown in a typical histogram taking an average of at least three experiments. Aconitase activity was assayed in J774.2 macrophages and HepG2 cells after treatment with APAP and DAS alone or in combination (6C). Activities of mitochondrial respiratory enzyme complexes I and IV were measured in freshly isolated mitochondria from APAP-treated cells using ubiquinone, and cytochrome c respectively as substrates as described in the Materials and Methods (6D). The values are expressed as mean ± SEM of three determinations. Asterisks (*) indicate significant difference (p≤0.05) from untreated control cells. # indicate significant difference (p ≤0.05) from APAP-treated group.

APAP treatment reduced the ATP levels drastically (40–80%) in HepG2 cells and macrophages (Fig 6A). Interestingly, DAS treatment recovered the ATP levels close to the control cells. The mitochondrial membrane potential, as determined by cationic dye membrane permeability, demonstrated an increased loss of membrane potential and DAS treatment has brought the potential close to that of the control untreated cells (Fig 6B). These results suggest that alterations in ATP production after APAP treatment are correlated with the disturbance in mitochondrial potential gradient. The mitochondrial matrix enzyme, aconitase, which is a sensitive marker for mitochondrial oxidative stress related dysfunction, was also found to be profoundly inhibited after APAP treatment which confirms our earlier observation [24–25]. DAS treatment recovered the enzyme activity close to the control untreated level (Fig 6C). However, a differential effect of APAP on the mitochondrial membrane bound respiratory enzyme complexes was observed in the macrophages and HepG2 cells. While activities of Complex I (NADH-Ubiquinone oxidoreductase) and terminal oxygen utilizing enzyme, Complex IV (cytochrome c oxidase) were negligibly disturbed in the HepG2 cells, there was marked inhibition in the activities of both enzymes in the APAP-treated macrophages (Fig 6D). DAS treatment alone had no appreciable effects on these enzyme activities. Only a marginal recovery of Complex I enzyme activity was observed after DAS treatment in APAP-treated macrophages.

### Effect of APAP on the Expression of Proteins

**Fig 7:**
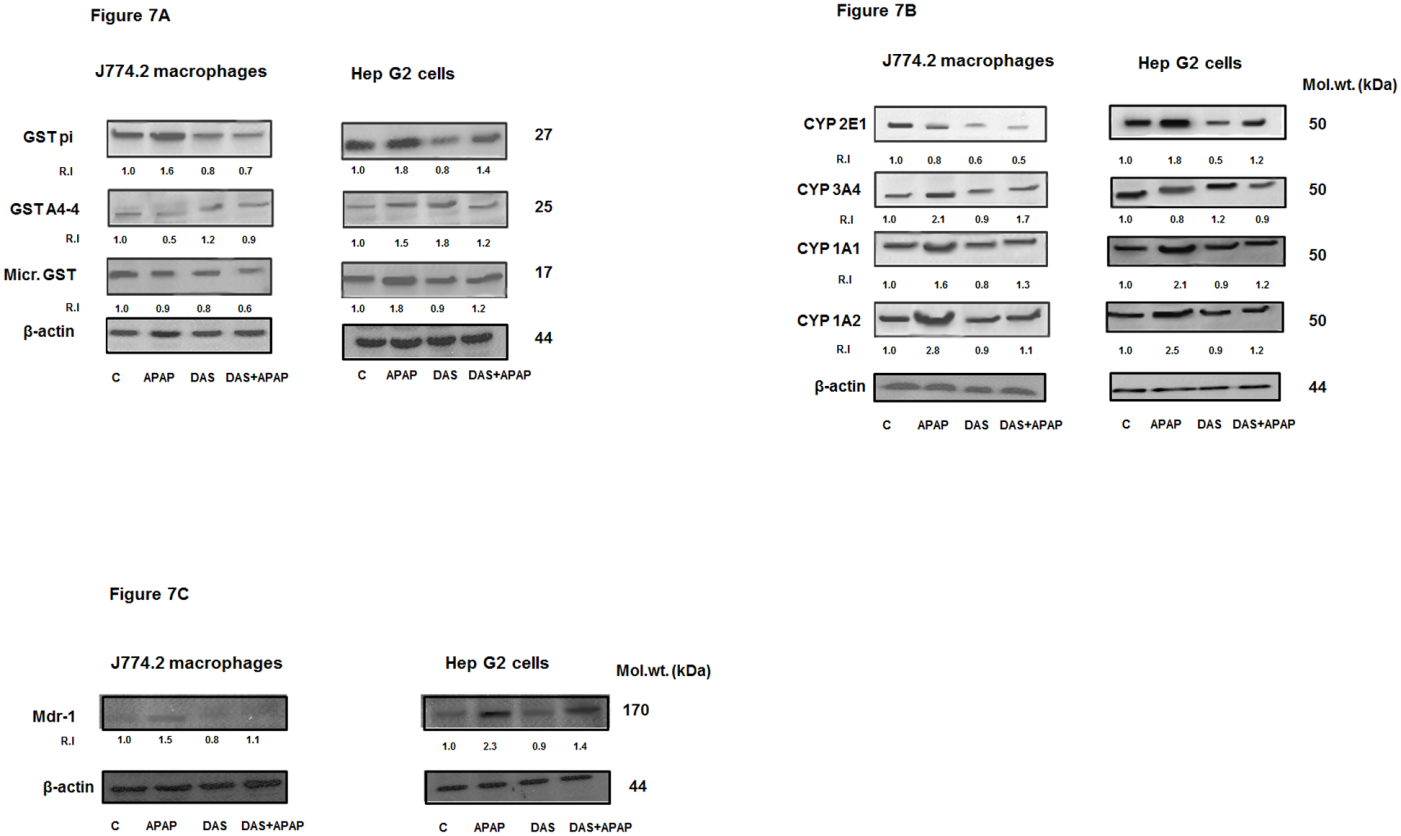
Expression of GST isoenzyme, CYP450 isoenzyme MDR1 proteins. Proteins (50μg) from PMS extract from APAP and DAS treated J774.2 macrophages and HepG2 cells were separated on 12% SDS-PAGE and transferred on to nitrocellulose paper by Western blotting as described in the Materials and Methods. GST pi, GSTA4-4 and microsomal GST (MGST1-1) proteins were visualized using specific antibodies against these proteins (Fig 7A). The expression of CYP isoenzymes was visualized using isoenzyme specific antibodies against CYP2E1, CYP3A4, CYP1A1 and CYP 1A2 (Fig 7B). MDR1 protein expression was measured using specific antibody against the protein (Fig 7C). Beta-actin was used as loading control. Representative Western blots from three experiments are shown. R.I gives the relative intensity of the protein compared to the control untreated cells as 1.0. Molecular weight is expressed in kDa.

Fig 7A shows the expression of cytosolic and microsomal GST proteins’ expression using isoenzyme-specific antibodies. APAP treatment resulted in almost 2-fold induction of GST pi protein in the macrophages as well as HepG2 cells. DAS pre-treatment brought the levels close to normal, more so in the macrophages. GSTA4-4, a member of the alpha GST family which conjugates lipid peroxidation product, 4-HNE, was slightly increased after APAP treatment in HepG2 cells. However, a significant reduction in the expression of GSTA4-4 was observed in the macrophages. This differential expression may be associated with the level of GSH metabolism and 4-HNE conjugation in these cell lines. On the other hand, the microsomal GST (MGST1-1) expression after APAP treatment was not significantly altered in macrophages while it was increased in the HepG2 cells. DAS treatment had minimal effects on the expression of microsomal GST.

Fig 7B shows the differential expression of various CYP450s in the macrophages and HepG2 cells after APAP and DAS treatment. While CYP2E1 protein expression was inhibited by APAP treatment, it was induced about 2 fold in HepG2 cells. DAS, a known inhibitor of CYP2E1 activity and expression, as expected, has markedly inhibited the enzyme expression in these cell lines. The pattern of expression of CYP3A4, on the other hand, was reversed. While a 2-fold increase after APAP treatment was observed in the macrophages, a marginal decrease in the expression was observed in HepG2 cells. The differential expression of CYP2E1 and CYP3A4 may be implicated with the preferential isoenzyme involved in the metabolism of APAP in macrophages and HepG2 cells and are in agreement of our previous reports [24–25]. CYP1A1 and CYP1A2 expression, on the other hand, was markedly increased in these cells and DAS treatment has brought the expression level close to the controls.

Fig 7C shows the expression of multidrug resistance glycoprotein, MDR1 in macrophages and HepG2 cells treated with APAP and DAS. Both cell lines exhibited an increased expression of MDR1 protein. However, the increased level of expression was more significant in HepG2 cells compared to macrophages. DAS treatment reversed the expression of the protein close to the controls. These results may suggest differential drug-conjugate exclusion which may be detrimental for drug-induced cytotoxicity and resistance in these cell lines.

## Discussion

Metabolism of APAP, a hepatotoxin, by a family of CYP450s, depletion of GSH, which conjugates the toxic metabolite of APAP and a burst of oxidative damages have all been implicated in APAP-induced toxicity [3,8,9,21]. Several antioxidants and activators of GSH pool, such as N-acetylcysteine, dithiothreitol, tea polyphenols, diallyl sulfide (DAS), taurine, melatonin, ascorbic acid, vitamin E, etc. have been shown to be beneficial in preventing APAP-induced toxicity [33,38–39]. However, the precise mechanism of cytotoxicity in different tissues and cellular systems is not clear. Macrophages have been implicated both in the attenuation as well as augmentation of APAP-induced responses [22, 23]. We have previously shown that APAP induced cytotoxicity and cell death in macrophages is associated with increased oxidative stress, alterations in GSH pool, oxidative protein carbonylation and activation of mitochondrial apoptotic signals [24, 25]. Our present study, using both macrophages and HepG2 cells has further confirmed that the cytotoxicity induced by APAP is indeed associated with increased oxidative stress and mitochondrial dysfunction. However, our results have also demonstrated that the macrophages appear to be more sensitive to APAP toxicity compared to HepG2 cells at the same dose and time point. This was demonstrated by increased DNA breakdown and apoptosis and depletion of GSH in the macrophages compared to HepG2 cells. These differential effects of APAP cytotoxicity in macrophages and HepG2 cells appears to be associated with the differential metabolism of APAP by CYP2E1 and CYP3A4 in these cells lines as well as differences in GSH metabolism by different isoenzymes of GST. As shown, macrophages exhibited a higher induction of CYP3A4 activity while a lower activation of CYP2E1 and GST A4-4 activities were observed. On the other hand, HepG2 cells exhibited higher activity of CYP2E1 and GSH-conjugating activities of CDNB by GSTpi and 4-HNE by GSTA4-4. CYP3A4 activity however, was, lower in HepG2 cells compared to the macrophages. On the other hand, the microsomal membrane bound GSH conjugating isoenzyme, was activated only in HepG2 cells but not in the macrophages. SDS-PAGE and Western blot analysis also confirmed the differential expression of CYP450 and GST isoenzymes in these cell lines. These results clearly suggest a differential mechanism of APAP activation by CYPs and conjugation by GST isoenzymes in the macrophages and HepG2 cells. Treatment with DAS, a garlic constituent with known CYP2E1 inhibitory activity, recovered the changes observed in CYP2E1 activity in HepG2 cells but not in the macrophages. On the other hand, DAS treatment alone had very little effect on the alterations in CYP3A4, CYP1A1 and CYP1A2 activities. GSH-Px activity in macrophages as well as in HepG2 cells was significantly lower in both cell systems suggesting increased oxidative stress and confirming our previously published results. DAS treatment marginally recovered the altered GSH-Px activity in both cell systems. Thus DAS acts not only via the inhibition of CYP2E1 activity but also as an antioxidant in these cell systems. There are numerous reports suggesting the involvement of DAS in protection against APAP toxicity due to the inhibition of CYP2E1, and increasing GSH metabolism, which is suggestive of an antioxidant effect for DAS [33, 40]. Interestingly, the expression of multi drug resistance protein, MDR1, which plays a role in drug exclusion and detoxification, including APAP [30–31, 41], was also markedly higher in HepG2 cells compared to macrophages which might be associated with increased efflux or detoxification of APAP from the HepG2 cells resulting in the development of some resistance in these cells, as seen by the low level of apoptosis and DNA damage in comparison to macrophages. Increased expression and activities of GST in APAP-treated HepG2 cells also support this observation.

We have further demonstrated that mitochondrial respiratory function and ATP synthesis was markedly affected by APAP treatment in macrophages as well as in HepG2 cells. The increased ROS production and apoptosis observed in APAP-treated macrophage J774.2 and HepG2 cells might be implicated with mitochondrial dysfunction in these cell systems. This was further confirmed by drastic inhibition of mitochondrial membrane potential, decreased activity of aconitase, a mitochondrial matrix enzyme and marker for oxidative stress, and by inhibition of the inner membrane respiratory enzymes complexes. This has been reported earlier using APAP and other NSAIDs. [24–29, 42]. Increased ROS production and mitochondrial oxidative stress also results in increased oxidative glutathionylation of respiratory complex I resulting in the inhibition of enzyme activity and disruption of energy homeostasis in APAP toxicity [43–46].

In summary, our results suggest that the differential cytotoxicity of APAP in macrophage J774.2 and HepG2 cells might be associated with their ability to metabolize and detoxify APAP differently in these cell systems. Also, there is some indication that MDR1 gene may also play a role in determining the sensitivity of macrophages and HepG2 cells towards APAP toxicity. These results may have long term implications to better understand the role of macrophages and HepG2 cells in the toxicology and pharmacology of APAP.
